# Johnny on the Spot-Chronic Inflammation Is Driven by HMGB1

**DOI:** 10.3389/fimmu.2019.01561

**Published:** 2019-07-11

**Authors:** Carolina M. Gorgulho, Graziela G. Romagnoli, Rosh Bharthi, Michael T. Lotze

**Affiliations:** ^1^Tumor Immunology Laboratory, Department of Microbiology and Immunology, Botucatu Institute of Biosciences, São Paulo State University, Botucatu, Brazil; ^2^DAMP Laboratory, Department of Surgery, Hillman Cancer Center, University of Pittsburgh, Pittsburgh, PA, United States; ^3^Department of Immunology, University of Pittsburgh, Pittsburgh, PA, United States; ^4^Department of Bioengineering, University of Pittsburgh, Pittsburgh, PA, United States

**Keywords:** HMGB1, chronic inflammation, autoimmunity, cancer, autophagy

## Abstract

Although much has been made of the role of HMGB1 acting as an acute damage associated molecular pattern (DAMP) molecule, prompting the response to tissue damage or injury, it is also released at sites of chronic inflammation including sites of infection, autoimmunity, and cancer. As such, the biology is distinguished from homeostasis and acute inflammation by the recruitment and persistence of myeloid derived suppressor cells, T regulatory cells, fibrosis and/or exuberant angiogenesis depending on the antecedents and the other individual inflammatory partners that HMGB1 binds and focuses, including IL-1β, CXCL12/SDF1, LPS, DNA, RNA, and sRAGE. High levels of HMGB1 released into the extracellular milieu and its persistence in the microenvironment can contribute to the pathogenesis of many if not all autoimmune disorders and is a key factor that drives inflammation further and worsens symptoms. HMGB1 is also pivotal in the maintenance of chronic inflammation and a “wound healing” type of immune response that ultimately contributes to the onset of carcinogenesis and tumor progression. Exosomes carrying HMGB1 and other instructive molecules are released and shape the response of various cells in the chronic inflammatory environment. Understanding the defining roles of REDOX, DAMPs and PAMPs, and the host response in chronic inflammation requires an alternative means for positing HMGB1's central role in limiting and focusing inflammation, distinguishing chronic from acute inflammation.

“*A ‘Johnny on the spot' is a man or youth who may be relied upon to be at a certain stated place when wanted and on whose assured appearance confident expectation may be based. It is not sufficient that an alert and trustworthy individual, to be thought deserving of the name ‘Johnny on the spot,' should restrict his beneficent activity to the matter of being at a certain place when needed. He must, in addition, render such service and attend to such business when there as the occasion may require, and such a ‘Johnny' must be on the spot not merely to attend to the business of others, but also to look after his own. Hence an individual who is prompt and farseeing, alive to his own interests and keenly sensible of means for promoting his own advantage is a ‘Johnny on the spot.”' Anonymous, April 1896*.

## Introduction

### DAMPs and Inflammation

In the context of sterile inflammation, damage-associated molecular pattern molecules (DAMPs) are considered the “signal 0” that mediates engagement of pattern recognition receptors (PRRs) in immune cells, leading to the activation (signals 1–4) and tissue integration and persistence (signal 5) of their effector functions. Polarization of the immune infiltrate relies on cues present at the site of injury, including DAMPs, as well as on its REDOX state (1). DAMPs include proteins such as heat shock proteins (HSP), histones and cytokines, but some non-protein molecules can also act as “danger signals” such as ATP, extracellular DNA or RNA, and mitochondrial DNA. The prototypic DAMP and focus of this review is the high mobility group (defined by mobility on electrophoretic gels) box 1 protein, HMGB1, a member of the extended HMG-box containing proteins. These molecules only bind with high affinity non-B-type DNA conformations that are either bent or unwound. HMG-box domains regulate transcription, replication, and DNA repair, all of which change chromatin conformation. The HMG family of proteins includes such molecules as the three superfamilies each containing a specified functional domain: 1) HMGA (AT-hook domain)-HMGA1 and 2; HMGB (HMG-box domain) including homologous functional proteins HMGB1, 2, 3, and 4; HMGN (nucleosomal binding domain) including HMGN1, 2, 3 and 4; the SRY, Sex-Determining Region Y Protein located on the Y chromosome; and the TCF/LEF Transcription Factors including Lymphoid enhancer-binding factor 1 and T Cell Transcription Factor 1 that are involved in the Wnt signaling pathway, recruiting beta-catenin as a coactivator to enhancer elements of genes (2).

HMGB1 was first described as a chromatin-associated molecule, but other properties related to its cytosolic roles, promoting autophagy and its extracellular roles, promoting inflammation, have since been described. HMGB1 is a non-histone nuclear protein that contains two lysine-rich DNA binding regions, A and B boxes, and an unusual C terminal acidic tail composed largely of aspartic and glutamic acid residues (3). As a component of nucleosomes, HMGB1 has attracted attention in the context of autoimmunity, a topic which will be discussed below.

Although other receptors for HMGB1 may be identified given its “sticky” properties and unusual acidic and basic domains, known receptors include Toll-like receptors (TLRs) 2, 4, and 9, as well as receptor for advanced glycation endproducts (RAGE), CXCR4/CXCR7 and T-cell immunoglobulin and mucin-domain containing-3 (TIM-3) (3, 4). Endothelial cells are early responders to tissue damage, and readily upregulate adhesion molecules and initiate neutrophil recruitment following HMGB1 binding to its receptors (1). Cells of the immune system also respond to HMGB1, which promotes pro-inflammatory cytokine release by macrophages including tumor necrosis factor alpha (TNF-α) and interleukin (IL)-6 and activation of dendritic cells (DCs) (1).

### Autophagy and Cell Death

Autophagy is a highly conserved multistep mechanism that allows maintenance of metabolic homeostasis in cells undergoing stress, i.e., nutrient and oxygen deprivation, infection or oxidative stress, among others. During autophagy, bulk portions of the cytoplasm or ubiquitinated targets, such as protein aggregates or damaged organelles, are enclosed in the autophagosome, a double-membraned structure which then fuses with a lysosome for cargo degradation and ATP generation, under tight regulation of a set of proteins collectively named the ATG (autophagy-related gene) proteins. Autophagy is therefore a survival mechanism which has, however, also been associated at extremes with cell death, specifically “autosis,” where dying cells display numerous autophagosomes in their cytoplasm, often as a prelude to apoptosis (5). Thus, in most instances of cell death, autophagy accompanies cell demise, but it is not necessarily its cause, with several components of the autophagic machinery being involved in processes related to cell death (6) including ferroptosis.

### Microvesicles and HMGB1

The efficiency in inducing an inflammatory response depends on the success of cellular communication, which in turn, depends on ligand-receptor interaction, wherein the ligand can be on the membrane of the stimulatory cell, free in the extracellular medium and in extracellular vesicles (EVs) (7, 8). In general, there are 3 different types of EVs, which are classified based on their size, biogenesis and composition (9, 10). The small vesicles originate in endosomal compartments called multivesicular bodies (MVBs), which fuse with the cell membrane to release their intraluminal vesicles to the extracellular medium, where they are identified as Exosomes (Exo), ranging from 30 to 150 nm (7, 11). Microvesicles/Microparticles (MPs) are vesicles ranging in size from 100 to 1,000 nm, budding directly from the plasma membrane (12). The last and largest type are apoptotic bodies, from 1,000 to 3,000 nm, originating from the cytoplasmic membrane of apoptotic cells (12). Interestingly, MPs and apoptotic bodies can be released from activated or apoptotic cells (13–15).

EVs are found in bodily fluids, such as amniotic fluid, breast milk, semen, plasma, saliva, nasal secretion, cerebrospinal fluid (CSF), ascites, synovial fluid, etc. (16). Several studies have stressed the importance of EVs in cell to cell communication because they are carriers of bioactive molecules, such as DNA, mRNA, miRNAs, lipids, adhesion molecules, cytokines, molecules involved with antigen presentation, autoantigens, DAMPs and PAMPs (7, 8, 17–21). Furthermore, during pathological processes, EV levels are increased, contributing to the development, progression and persistence of inflammatory and autoimmune diseases and cancer (19, 22–25).

## Compartmental Roles of HMGB1

### Nuclear

**Figure 1:**
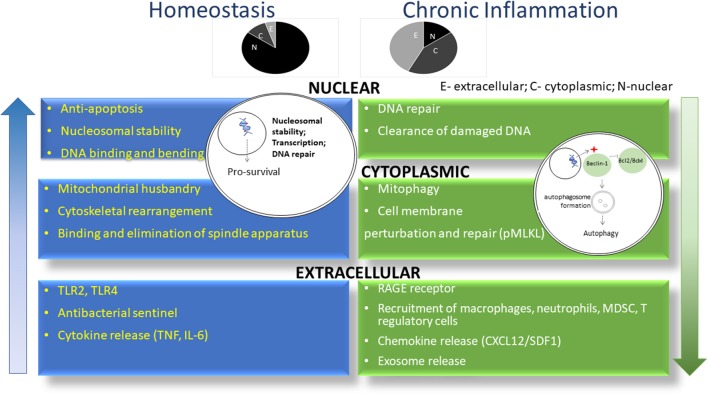
HMGB1 exerts differential roles in homeostasis and chronic inflammation. As shown in blue, HMGB1 is predominantly located in the nucleus of cells in the setting of homeostasis, where it promotes nucleosomal stability and facilitates access of transcriptional factors to DNA. In chronic inflammation (green), HMGB1 leaves the nucleus to drive autophagy in the cytoplasm and act as a DAMP in the extracellular *milieu*. Shown above are the relative proportion of HMGB1 in individual compartments during homeostasis and chronic inflammation. N, nucleus; C, cytoplasm; E, extracellular.

The various functions of HMGB1 are highly context dependent. In the nucleus of homeostatic cells (Figure 1), HMGB1 is loosely associated with DNA, mediating its replication, transcription, recombination, repair and overall stability (1, 3). For example, HMGB1 preferentially binds damaged DNA following ionizing radiation, treatment with platinum or other DNA damaging chemotherapy and undergoes post-translational modifications to recruit and directly interact with other proteins involved in DNA repair, such as p53 (26, 27). Additionally, HMGB1 and p53/p73 interact in the nucleus, promoting access to their transcriptional complexes on DNA. p53 regulates the subcellular localization of HMGB1, inhibiting its translocation to the cytoplasm and *vice-versa* (28). This has proven to be a pivotal mechanism for balancing subsequent apoptotic and autophagic processes. HMGB1 contributes to chemoresistance by exporting p53 out of the nucleus and into the cytoplasm, where it undergoes autophagic degradation (29). HMGB1 is important for all of the DNA repair enzymes in the nucleus (30). There it serves as a “Jack-of-all-trades” in addition to its “Johnny-on-the-spot role,” facilitating the nucleotide excision repair (NER) pathway, the base excision repair (BER) pathway, the mismatch repair (MMR) pathway, the non-homologous end-joining (NHEJ) pathway, and V(D)J recombination in T and B cells.

Unlike histones, which are associated with maintenance of chromatin structure, HMGB1 can both compact and destabilize chromatin, facilitating access to numerous transcription factors (26). HMGB1 has previously been associated with regulation of transcription factors related to cell death (31), hormonal (32), and immune responses (33). HMGB1 contributes to liver tumorigenesis by positively regulating the expression of yes-associated protein, which forms a complex with hypoxia-inducible factor 1α to drive hepatic cells to acquire a glycolytic metabolic profile (34). Additionally, HMGB1 controls transcription of HSP beta-1/HSP27, essential for mitochondrial quality control, resultant mitophagy, and thus maintenance of metabolic homeostasis (35).

### Cytoplasmic

In the face of stress, HMGB1 is actively translocated from the nucleus to the cytoplasm through oxidation of the cysteine encoded at position 106 as well as post-translational modifications posited including ADP ribosylation (36) and acetylation (37). There, it frees Beclin-1 from Bcl-2/BCLxL interaction sites, promoting formation of autophagosomes. Importantly, binding of HMGB1 with Beclin-1 is dependent on disulfide bond formation between cysteines 23 and 45. Treatment of MEFs and tumor cells with autophagy inhibitors prevents LC3 puncta formation and HMGB1 translocation. Knockdown of Atg 5 has a similar effect (38). Thus, HMGB1 translocation to the cytoplasm induces autophagy but the occurrence of autophagy also regulates HMGB1 translocation.

Autophagy is essentially a pro-survival cellular mechanism. Given the close relationship between HMGB1 and autophagy, there has been extensive research into the role of HMGB1 and chemoresistance in various types of tumors and classes of drugs, which will be discussed below.

### Extracellular

HMGB1 is actively secreted to the extracellular milieu by components of the immune system, platelets and endothelium following infection and exposure to inflammatory mediators (39). Oxidative stress-mediated autophagy is also a trigger for HMGB1 active release (38). As a leaderless molecule, HMGB1 undergoes lysosomal exocytosis and passive diffusion, given its polybasic tracts and protein transduction domain like properties, to gain access to the extracellular space (3, 40). It is also released from dying cells, both necrotic (with loss of membrane integrity) and late apoptotic, partly as a result of a cell's failure to undergo efferocytosis (41–45). Apoptotic cell-derived HMGB1 usually does not possess immune activating properties due to its oxidized state (39). Once out of the cell, HMGB1 can act as a DAMP through direct binding with its receptors or it can interact with other molecules including IL-1, DNA, RNA, miRNA, LPS and nucleosomes, dictating the range of different receptors that can be bound and the consequent biologic effect. The oxidative state of HMGB1 determines its roles as a chemokine, when reduced, partnering with CXCL12 to attract leukocytes, or as a cytokine, when partially oxidized, forming disulfide bonds and binding TLR4. Cell fate also seems to be regulated by HMGB1with reduced forms of HMGB1 decreasing the cytotoxicity of various chemotherapeutic agents by inducing autophagy whereas its oxidized form has the opposite effect, contributing to cell death (46).

## HMBG1 and Autoimmunity

### HMGB1 and Human Disease

HMGB1 is upregulated in the serum/plasma of patients with various autoimmune disorders including vessel vasculitis, systemic lupus erythematosus (SLE) and rheumatoid arthritis (RA) (47). Furthermore, patients with active disease present with higher levels of serum/plasma HMGB1 than those with inactive disease (47), highlighting its importance as a mediator of autoimmunity.

#### Systemic Lupus Erythematosus

SLE is an autoimmune disease characterized by the production of antinuclear antibodies, with nucleosomal antigens being the main targets, which induces systemic, potentially life-threatening symptoms (48, 49). In this disease, a high rate of cell death or persistence of non-cleared apoptotic cells lead to a large load of nuclear autoantigens being released in tissues or the blood, triggering autoimmunity (50). It has been speculated that non-cleared apoptotic cells can assume characteristics of “secondary necrotic cell death,” leading to HMGB1 release to the extracellular milieu and driving autoimmunity further, but whether this effect relies on HMGB1 alone remains to be clarified. Accordingly, genetic defects in the complement component C1q, an opsonin involved in apoptotic cell clearance, are associated with SLE in humans (51, 52). Neutrophils from patients with SLE are more prone to undergo NETosis, a form of cell death where a mesh of chromatin along with other nuclear components and antimicrobial effectors (termed NET, neutrophil extracellular trap) is externalized (53, 54). It has been proposed that the presence of HMGB1 in NETs can prevent their clearance through inhibition of DNAse I activity, leading to lupus nephritis, a complication of SLE (55, 56). Moreover, there can be an increase in HMGB1 serum levels from patients with pedriatic lupus nephritis in comparison to SLE patients without renal involvement (57). Defects in NET degradation have also been associated with other autoimmune disorders characterized by the presence of autoantibodies (48, 58, 59) but the role of HMGB1 in these settings has not been thoroughly explored. Dendritic cells (DCs) preferentially present antigens from NETotic neutrophils (60) and HMGB1 present in NETs released by neutrophils from pedriatic SLE patients up-regulate type I interferon production by tissue plasmacytoid DCs, which further stimulates the release of NETs and aggravates the disease (61). Macrophages can also respond to HMGB1, upregulating the production of inflammatory cytokines TNF-α and IL-6 both *in vitro* and *in vivo* (62, 63).

#### Rheumatoid Arthritis

In rheumatoid arthritis (RA), inflammation and hyperplasia of the synovial membranes can be worsened by angiogenesis and consequent increase in the influx of inflammatory infiltrate. Fibroblasts from RA patients readily enhance HIF-1α expression following HMGB1 treatment via TLR4 engagement and signaling through NF-κB (64). Furthermore, conditioned medium from HMGB1-treated fibroblasts from RA patients induce endothelial cell tube formation via VEGF release. HMGB1 neutralization attenuates symptoms of experimental arthritis, with significant lower expression of HIF-1α and VEGF *in vivo*. The anti-inflammatory drug cilostazol reduces these angiogenic effects of HMGB1 (65). HMGB1 is upregulated in the spinal cord of arthritic mice and intra-thecal administration of anti-HMGB1 Ab reverses mechanical hypersensitivity in these animals, a symptom that is attributed to the action of disulfide HMGB1 alone (66). Monocytes from patients with active RA require lower amounts than healthy controls of HMGB1 to acquire a migratory phenotype dependent on CXCL12, a chemokine known to form a heterocomplex with HMGB1 in the extracellular milieu to drive inflammation (67). Interestingly, high levels of the enzymes thioredoxin and thioredoxin reductase (which have previously been associated with disease severity) are found in the plasma of RA patients and maintain the reduced status of HMGB1, its ability to bind CXCL12 and therefore exert inflammatory activity. Another important partner of HMGB1 in the pathogenesis of RA is LPS, which activates synovial fibroblasts to produce inflammatory cytokines, matrix-metalloproteinases, increase autophagic flux and decrease apoptosis (68, 69). Methotrexate, a drug commonly used in the treatment of patients with RA, decreases HMGB1 levels and hyperplasia in the synovial tissue. HMGB1 knockout in fibroblasts from these patients renders them less proliferative and invasive (70). However, methotrexate treatment can also stimulate autophagic flux in RA fibroblasts via an HMGB1-Beclin1-dependent fashion, culminating in chemoresistance (71).

#### Multiple Sclerosis

Multiple sclerosis is an autoimmune demyelinating disease characterized by high concentrations of extracellular HMGB1 and its receptors, RAGE and TLR's, in patients' plasma (72) or cerebrospinal fluid (73). Additionally, there is enhanced cytosolic expression of HMGB1 in cells within MS lesions. In experimental autoimmune encephalomyelitis (EAE), HMGB1 neutralizing antibody has both prophylactic and therapeutic effects, preventing oligodendrocyte loss and CD4^+^ T cell recruitment to the central nervous system of treated mice (74). Some of the anti-inflammatory drugs that are currently being studied for the treatment of EAE act through inhibition of HMGB1, decreasing inflammatory infiltrate, production of cytokines, neuronal damage, activation of cells in the central nervous system and overall diminishing the severity of the disease (75, 76). In experimental autoimmune myocarditis, silencing of HMGB1 in macrophages prevents their polarization to the M1 phenotype following activation with LPS and prevents activation of the NF-κB pathway (77). Furthermore, i*n vivo* silencing of HMGB1 prevented M1 macrophage infiltration and protected the cardiac tissue of treated mice. Fingolimod (Gilenya), the first FDA-approved oral disease-modifying drug for the treatment of MS, reduces serum levels of HMGB1 in patients which may be a suggested marker for clinical relapse (78). HMGB1 can be found in the nucleus of astrocytes and macrophages during the progression of EAE but in neurons, HMGB1 is found mainly in the cytoplasm during the onset phase, indicating that different cell types and subcellular localization of HMGB1 can contribute to pathology in this setting (79).

#### Psoriasis Vulgaris

Psoriasis vulgaris (PV), a dermatological disease initially categorized as a hyperkeratotic disorder, has more recently been redefined as to include an immune-mediated chronic inflammatory aspect to its pathophysiology, involving systemic activation of T cells and production of inflammatory cytokines, including HMGB1 (80–82). A role for the reprogramming of Tregs into IL-17 producing cells in psoriatic lesions has also been reported (83). Serum concentrations of HMGB1 in PV patients are higher than healthy controls and have been found to correlate with disease severity according to the Psoriasis Area Severity Index (80, 81). Furthermore, patients undergoing treatment with TNF-α blockade, fumaric acid and methotrexate, but not IL-12/IL-23 inhibitors, presented with a reduction in serum levels of HMGB1 (80). Skin lesions from PV patients show increased positivity for extranuclear HMGB1 (81, 84) and in patients with severe PV, healthy skin biopsies also show such an increase (84). It has also been reported that circulating CD8^+^ T cells as well as Tregs from PV patients have increased expression of HMGB1's receptor RAGE, further indicating the involvement of T cells in the onset and progression of disease and suggesting possible therapeutic targets. However, the defined role for HMGB1 in PV has not been fully elucidated. There is evidence that autophagy limits keratinocyte inflammatory responses and since HMGB1 is a known inducer of autophagy, one can speculate that the higher levels of HMGB1 in the serum of patients with severe disease could be a regulatory mechanism rather than a driver of inflammation (80, 85).

#### Atopic Dermatitis

Atopic dermatitis (AD) is a chronic inflammatory skin disease characterized by high levels of serum IgE and pruritic skin lesions that are infiltrated by mast cells, eosinophils, macrophages, DCs and T cells, particularly those of the Th2 profile, with cytokines like IL-4, IL-13, and IL-31 playing important roles in its pathophysiology (86). In a murine model of 2,4-dinitrochlorobenzene (DNCB)-induced AD, HMGB1 and RAGE were found in high concentrations within the lesions of DNCB treated mice, along with higher levels of TNF-α, IL-6, and phosphorylated NF-κB, all of which were reduced after treatment with glycyrrhizin, a compound that targets HMGB1 (87). Interestingly, while in healthy controls HMGB1 was confined to the nucleus, in AD lesions it was found in the cytoplasm. Moreover, the authors show that HMGB1 can activate and recruit mast cells *in vivo*, thus contributing to the pathogenesis of AD, an effect which was also abrogated by glycyrrhizin. In human samples, the highest extracellular HMGB1 concentrations were found in skin lesions from AD patients, followed by PV patients and then healthy controls (88). The same pattern is observed when comparing HMGB1 expression on immune cells that infiltrate the lesions or reside in normal skin. Additionally, epithelial cells from AD samples present with more nuclear p65 than PV samples and healthy controls, suggesting an HMGB1-NF-κB axis that may be at play in AD. This axis was also explored *in vivo*, in a study where treatment with the flavonoid quercetin inhibited HMGB1 translocation from the nucleus to the cytoplasm in lesions and decreased levels of RAGE, TLR-4, and nuclear NF-κB proteins in tissue homogenates (89). In an organotypic human epidermis model, treatment with HMGB1 or IL-4 downregulates the expression of several proteins related to skin barrier function and increases production of IL-33, an inflammatory cytokine known to be upregulated in skin lesions of AD patients (90). Furthermore, HMGB1 stimulation of keratinocytes also impairs epidermal growth and maturation *in vitro*. These results suggest that HMGB1 could also act on AD through disrupting homeostasis of keratinocytes and impairing the skin's barrier function. In addition to loss of skin barrier function, AD can be accompanied, amongst other clinical features, by high levels of circulating IL-17 and IL-23. Recently, it has been reported that HMGB1 serum levels positively correlate to disease severity, serum IgE, IL-17, and IL-23 concentrations and inversely correlate to circulating IL-10 levels (91).

#### Allergic Rhinitis

Allergic rhinitis is a very common disorder caused by IgE-mediated nasal inflammation which is further propagated by the cytokines produced by the immune infiltrate (92). HMGB1 levels in the nasal lavage of children with untreated rhinitis is significantly higher that healthy controls and correlates with severity of disease (92, 93). Similar results were reported with patients with chronic rhinosinusitis (94). As with AD, glycyrrhizin also seems to attenuate the symptoms of allergic rhinitis by targeting HMGB1 and reducing its concentration in the nasal fluid of treated patients (93). The authors also show that glycyrrhizin selectively kills eosinophils isolated from peripheral blood of healthy donors *in vitro*, which could also account for its effects *in vivo*. In an OVA-induced model of AR, treatment with SIRT1 had both systemic and local anti-inflammatory effects (95). Interestingly, the drug significantly decreased the levels of HMGB1 found in the nasal mucosa *in vivo* and reduced signaling through the HMGB1-NF-κB pathway *in vitro*.

### Microvesicles and Autoimmunity

In autoimmunity, platelet EVs seem to influence the course of disease, being present in high levels in patients with RA (96–98), SLE (98, 99), Grave's disease (100) and systemic sclerosis (101). In RA, EVs can act through several different pathways, presenting antigens to the immune system, degrading extracellular matrix, carrying miRNA and autoantigens, such as citrullinated proteins, resulting in induction of inflammation, as well as perpetuating it by formation of bioactive immunocomplexes (ICs) (23, 98).

RA patients who are seropositive for CCP^+^/RF^+/−^ (anti-cyclic citrullinated peptides and rheumatoid factor, respectively) present systemic high levels of inflammatory cytokines and CD14^+/−^/CD16^+^ monocytes (called intermediate profile) in comparison to their seronegative counterparts (96). Interestingly, these patients have high levels of HMGB1^+^ EVs in both the blood and synovial fluid. Systemic EVs associate with IgG and IgM to form ICs (EV-ICs), which are internalized by patient-derived mononuclear phagocytes *in vitro* and induce IL-1β, IL-6, and TNF-α production. HMGB1^+^ EVs may thus have a role in the maintenance of systemic inflammation seen in seropositive patients, through their action on monocytes and/or DCs. Moreover, HMGB1 alone or in EVs can also associate with autoantigens (102), which in the extracellular microenvironment can act as a potent inflammatory cytokine, inducing production of other pro-inflammatory factors by monocytes (103). Not only monocytes, but also neutrophils can be influenced by EV-ICs in RA patients, since they induce leukotriene production by these cells *in vitro* (97).

Similar to what happens in RA, microparticles from SLE patients form ICs (IgG and IgM), and MPs associated with IgG are correlated with active disease (104). ICs directly influence the course of SLE, as they lead to deposition in tissues such as kidneys or skin and activate cells of the immune system to induce lesions (105). Contributing to the progression of the disease, phosphatidylserine negative MPs have significant expression of HMGB1, tissue factor (TF) and vascular cell adhesion protein 1 (VCAM-1) (99), proteins that are involved with inflammation, thrombotic events and cardiac disorder, phenomena that are characteristic of SLE. Moreover, these MPs show a relationship with the decrease of cystatin C, reducing renal function, as well as increasing expression of TNF receptor, which is associated with active nephritis (106).

Systemic sclerosis associated with pulmonary arterial hypertension (101) is another multisystemic disorder with high levels of HMGB1 in MPs, reinforcing the notion that vascular damage and inflammation are prominent in these patients.

The high levels of anti-TSH receptor in Grave's disease indicate intense immune system activation (107) with inflammatory response. This phenomenon seems to reflect in serum MPs of the patients, presenting high expression of pro-inflammatory molecules such as HMGB1, P-selectin, and CD40L (100). Furthermore, the authors demonstrate the presence of monocyte-derived MPs with double positivity to HMGB1 and SYTO 13 (which stains RNA/DNA), indicating they originate from apoptotic cells. After antithyroid treatment, patients present less HMGB1^+^/SYTO 13^+^ MPs, but not equal to the levels of healthy donors.

Platelet and endothelial MPs from relapsing-remitting MS patients increase endothelial barrier permeability (108), besides attracting leucocytes, extending the inflammation of the central nervous system and exciting endothelial cells to produce TNF-α (109). Since EVs reflect their cell of origin, which are directly influenced by the microenvironment, it is possible to speculate that HMGB1 is present in these vesicles, even though, this has not been thoroughly investigated. HMGB1^+^ MPs are eligible candidates for participation in the pathogenesis of inflammatory bowel diseases, since EVs isolated from colonic luminal fluid induce a pro-inflammatory profile on macrophages and epithelial cells *in vitro* (110).

## HMGB1 and Cancer

### Cancer Is the End Stage of Chronic Inflammation

Pathologists distinguish between chronic (>30 days) and acute (<30 days) inflammation based on the presence and prevalence of lymphocytes (chronic) or neutrophils (acute). DAMPs and PAMPs promote the initiation of an immune response driven by tissue injury or pathogens respectively, which recruits innate inflammatory cells. Adaptive immune cells start to replace innate cells within 3–10 days. HMGB1 plays an important and decisive role in regulating the phenotype, maturity, and behavior of DCs. Firstly, HMGB1 is a chemoattractant for immature DCs (111). HMGB1, specifically the B box motif, stimulates DCs to mature via TLR4 signaling (111–113). This has been shown by the presence of co-stimulatory surface molecules CD80, CD86, and HLA-A, -B, -C, as well as various other marker molecules that demonstrate maturation of DCs (111). As DCs mature, they also secrete inflammatory cytokines, such as IL-6,−8,−12,−1α, TNF, and RANTES (112). Furthermore, signaling via the p38-MAPK pathway, HMGB1 drives DC to express CD83 and secrete IL-6 (112). HMGB1 can also promote secretion of IL-23, another IL-6/IL-12 family member, in bone marrow-derived DCs, which then in turn promotes Th17 cell differentiation (114). In effect, HMGB1 promotes an inflammatory microenvironment through its signaling and stimulation of DCs.

In the context of cancer, HMGB1-mediated inflammation is certainly significant. In the presence of HMGB1, DC receptors CXCR3 and CCR5 are upregulated in the tumor microenvironment of lung cancer tissue, enhancing migration of DCs (5). HMGB1 originating from tumor cells promote DC recruitment into the tumor microenvironment. HMGB1 and IFN-γ from CD8^+^ T cells are positively correlated, forming a positive network: IFN-γ promotes HMGB1 secretion in tumors, which in turn promotes CD8^+^ T cells to secrete more IFN- γ (115). Additionally, IFN- γ stimulated tumor cells to produce CCL5, CXCL10, and CXCL11, which further supports the notion that DCs will be attracted toward tumor (115). HMGB1 attraction of DCs to an inflamed site, may also promote tolerization without additional PAMPs or other TLR signals.

With chronic release of DAMPs or PAMPs, professional antigen presenting cells (both recruited inflammatory DCs as well as tissue resident CD103+/CD141+ DCs) promote integration and maturation of the inflammatory response, promoting cells of the so-called “adaptome.” The adaptive immune system is the “best doctor” in wartime for both diagnosing and treating diseases, integrating five elemental, highly networked lymphoid cells that both support and counter-regulate each other: NK cells, NKT cells, αβ T-cells, γδ T-cells, and B cells expressing an IgH and κ or λ light chains. It carries out these tasks with unmatched precision with the help of rearranged T and B cell receptors, our most diverse set of expressed and rearranged genes, fundamentally distinguishing one individual from another. This autologous potential receptor diversity, ranging from 10^15^ to 10^25^ for each chain of the rearranged receptors, contains only two chains expressed in each cell. The immune repertoire is the sum of the individual clonotypes within one chain, including individual CDR3 sequences.

The primary role of HMGB1 within the cell is to regulate transcription in the nucleus. However, as mentioned previously, when a cell undergoes necrosis or necroptosis, HMGB1 is released, acting as a DAMP and promoting inflammatory pathways. While HMGB1 plays a role in sustaining chronic inflammation and promoting wound healing, it can also trigger pathways which promote tumor growth including angiogenesis, reparative epithelial proliferation, efferocytosis by recruited macrophages and inhibition of immune effectors mediated by myeloid derived suppressor cells (MDSCs) and T regulatory cells (Tregs) (116). This has led to the notion that chronic inflammation, a wound healing phenotype, and cancer progression are closely related.

Indeed, HMGB1 contributes to immune escape and tumor progression *in vivo* by stimulating the proliferation of MDSCs as well as increasing their T cell-inhibitory properties (117, 118). Lewis lung carcinoma cells treated with resveratrol express less HMGB1 and induce less MDSC mobility when co-cultured, an effect that was partially reversed by treatment with exogenous recombinant HMGB1 (119). The supernatant from MCF-7 breast tumor cells is rich in HMGB1 and skews bone marrow progenitors into MDSCs, a phenomenon which is abrogated by treatment with ethyl pyruvate or anti-HMGB1 antibody (120). Serum levels of HMGB1 and the frequency of MDSCs are correlated and increased in breast cancer patients, MDSCs cultured under starvation conditions and in the presence of ethyl pyruvate do not upregulate autophagic markers and have their viability drastically reduced (121). HMGB1-induced autophagy not only promotes tumor growth by directly enhancing tumor cell survival, but also by boosting the immunosuppressive nature of the TME by perpetuating regulatory cells. HMGB1 can also act on MDSCs to facilitate metastatic dissemination. HMGB1-mediated recruitment of MDSCs to the peritoneal cavity of mice can increase their metastatic burden post-resection surgical extirpation (122). MDSCs are also associated with immunosuppression after trauma and sepsis. Accumulation of MDSCs in the spleen of mice following trauma is dependent on tissue-derived HMGB1 (123).

Tregs are another major population at play in chronic inflammatory environments as well as within the TME. In autoimmunity, there are many reports demonstrating that neutralizing antibodies or drugs that directly target HMGB1 can reduce inflammation and stimulate the development of Tregs, ameliorating symptoms of diseases like type 1 diabetes (124), autoimmune thyroiditis (125), and graft-vs.-host disease (126). In the chronic inflammatory setting, HMGB1 levels in the serum of atherosclerotic patients is increased, which negatively correlates with the Treg/Th17 ratio, promoting progression of the disease (127). In chronic HBV infection, HMGB1-mediated autophagy is important for Treg survival and functionality (128).

In cancer, HMGB1 can be associated with differentiation of Tregs, recruitment to the TME and enhancement of suppressive features. Tumor cell-derived HMGB1 increases the absolute numbers of Tregs in the spleen and draining lymph nodes of tumor-bearing mice, while also stimulating Tregs to produce IL-10 and suppress T cell activation (129). Co-culture of PBMCs with the neuroblastoma cell line SK-N-SH and its supernatant induces differentiation of FoxP3^+^ CD4^+^ T cells. The suppressive function of these cells was not tested. This effect is abrogated by HMGB1 neutralization (130). An HMGB1-TSLP (thymic stromal lymphopoietin) axis has been demonstrated, where tumor-derived HMGB1 and TSLP enhance DC-mediated activation of Tregs, a phenomenon that was dependent on the presence of the TSLP receptor on DCs (131). Furthermore, intratumoral inhibition of HMGB1 boosted T-cell dependent antitumor immune response *in vivo*. In head and neck cancer patients, HMGB1 acts as a chemokine for Tregs and enhances their suppressive capacity, with both tumor-infiltrating and circulating Tregs expressing the HMGB1 receptors TLR4 and RAGE (132). In breast cancer patients, the presence of intranuclear HMGB1 in tumor cells (that is, non-secreted HMGB1) is a favorable prognostic factor and negatively correlates with infiltration of Tregs or tumor-associated macrophages (133).

Chronic inflammation in the setting of cancer arises with cell death and release of DAMPs from necroptotic and necrotic cells. These cells break down and end up releasing HMGB1 into the extracellular space along with other DAMPs, including ATP, histone H1, S100 molecules, heat shock proteins, DNA, and RNA. HMGB1, often coupled with free-nucleotide-containing molecules, activates PRRs, which then activate inflammatory and wound healing pathways. These downstream effects provide the scenario that eventuates in the cycles of cell death, DAMP release, and reparative proliferation, conditions that ultimately lead to chronic inflammation (116).

Individual PRRs activated by HMGB1, such as TLRs, also contribute to tumor progression. TLRs play significant roles in metastasis promotion, immune evasion, and nascent and perpetual neoangiogenesis (116). This promotes tumor replication, emergent genomic instability, and Darwinian selection in promoting its ability to develop and grow.

Once a tumor has been established, it can continuously produce DAMPs and release them into the surrounding tumor microenvironment (116). Although somewhat higher levels of HMGB1 are expressed in some tumors, its localization to the cytosol and extracellular release associated with emergent autophagy more often dictates the outcome (in addition to receptor expression including RAGE and the TLRs) (134). The emergent tumor now contributes to releasing DAMPs into the local area, thereby promoting both cancer development and inflammation. These two pathways play off each other, causing more and more HMGB1 to be released and leading to a positive feedback of both pathways. Therefore, chronic inflammation directly initiates cancer. This apparent positive feedback stems from DAMP release and emergent secondary genomic instability, showing that chronic inflammation and cancer are intertwined.

### Microvesicles and Cancer

Inflammation is a core hallmark of cancer (135), which may be related with initiation, promotion, invasion and metastasis (136, 137). In this context, EVs, being mediators of cellular communication, have pivotal roles in facilitating progression, metastatic niche formation and metastases, driving tumor-promoting inflammation (136–138). Tumor EVs, such as Exo, may contain pro-inflammatory molecules, such as HSP, PGE-2, and HMGB1 (25, 139). The paracrine or autocrine action of HMGB1 can participate on chemoresistance to drugs such as oxaliplatin and 5-fluorouracil (5-FU), by the induction of autophagy (140) and repair of DNA adducts. Interestingly, culture supernatant of chemoresistant cells are rich in HMGB1. Although not the focus of that study, it is possible to speculate that at least part of the measured HMGB1 could be found within EVs in the medium.

Tumor-derived exosomes containing HMGB1 can modulate the microenvironment through subversion of the immune response. Patients with gastric cancer have high plasma and tumor tissue HMGB1 expression and this correlates with poor prognosis. Furthermore, Exo from culture supernatant and tumor tissue induce a pro-tumor profile in neutrophils, which is dependent on expression of exosomal HMGB1 (25). This recognition occurs via signaling through TLR4, activating NF-kB and STAT3, which confers on neutrophils resistance to apoptosis, increased formation of autophagosomes and production of pro-inflammatory cytokines (IL-1β, IL-6 and TNF-α) and molecules involved with migration and invasion, such as oncostatin M (OMS), MMP9, and VEGF. These phenotypic changes induce increased gastric tumor cell migration *in vitro*.

HMGB1 is highly expressed in the tumor tissue, often with enhanced cytosolic translocation, in almost all cancers including breast, colorectal, pancreatic, and hepatocellular carcinoma (134, 141, 142). Exosomal HMGB1 of human liver cancer cell lines induces B regulatory (Breg) differentiation, proliferation, and activation in healthy donors (143). This differentiation occurs through engagement of TLR2/TLR4 in B lymphocytes, which express TIM (T cell Ig and mucin domain)-1 membrane glycoprotein. Additionally, TIM-1^+^ Breg cells in contact with CD8^+^ T lymphocytes induce suppression, decrease proliferation and TNF-α and IFN-α expression. TIM-1^+^ Breg are excellent producers of IL-10, presenting a correlation with increased recurrence, decrease of overall survival and disease-free survival of hepatocellular carcinoma patients (143), which together are associated with a poor prognosis.On the other hand, treatment of cervical cancer cell lines with 5-Aminolevulinic acid photodynamic therapy (ALA-PDT) can increase HMGB1 content in tumor-derived Exo, which was found to be beneficial to the activation of APCs, inducing secretion of IL-6, IL-12, IL-18, IFN-γ, and TNF-α *in vitro* (144).

Thus, extracellular vesicles are efficient messengers, systemically or locally, which reflectthe phenotype of the cell of origin, being able to also act as biomarkers. They are loaded with nucleic acids, lipids and protein, including HMGB1 and other DAMPs, inducing changes that contribute with progression of autoimmune diseases and cancer, by increasing the inflammatory response. However, there is also evidence pointing to the fact that Exo can carry immune-activating molecules and that HMGB1 could be one of them.

### HMGB1 Expression in Human Cancers and Its Prognostic Value

Both systemic and local HMGB1 evaluation demonstrate that its expression frequently accompanies advanced disease stages and poorer prognosis in most epithelial tumors including lung, colorectal (CRC) and pancreas (Table 1). Chemotherapy can induce higher levels of circulating HMGB1-containing nucleosomes in lung cancer patients, a factor predictive of low differentiation of tumor cells and a more invasive phenotype (176). In esophageal cancer patients, neoadjuvant radiation and chemotherapy correlate with increased serum levels of HMGB1 and pulmonary complications (169). Similarly, in patients undergoing heated intraperitoneal chemotherapy, higher rates of HMGB1 released into the extracellular space positively correlate with complication rates (177). Following radioembolization therapy in CRC patients, high serum levels of HMGB1 are a predictive factor for failure to respond to treatment while both pre and post therapeutic high HMGB1 correlate with poor overall survival (167). There is also evidence linking HMGB1 expression in tumors to recruitment of immune infiltrate [although the opposite phenomenon has also been observed (158, 159)] associated with better activation of anti-tumor immune responses (149, 150, 170). The absence of correlation between HMGB1 in the tumor microenvironment and immune infiltrate has also been reported in both breast and esophageal cancers (178). In a recent study that evaluated The Cancer Genome Atlas (TCGA) of the pancreatic adenocarcinoma dataset, HMGB2, but not HMGB1, was found to have predictive prognostic value, with high expression associated with worse outcome (179). In a pancreatic ductal adenocarcinoma (PDAC) cohort, high serum HMGB1 levels proved to have diagnostic potential, besides positively correlating with stage of disease, presence of metastasis, tumor size and worse prognosis (174). Subcellular localization of HMGB1 can be of importance regarding disease outcome. In stage III CRC patients, localization of HMGB1 to the nucleus correlates with better recruitment of CD45^+^ cells and survival rate, whereas co-localization to both nucleus and cytoplasm has the opposite effect (158). In PDAC patients, the presence of nuclear HMBG1^+^/HMGB2^−^ tumor cells correlate with significantly shorter postoperative survival (172). However, low nuclear HMGB1 expression can also be associated with shorter median survival time in pancreatic (173) and with poor prognosis in breast cancer (133).

**Table 1:** HMGB1 expression and subcellular localization in epithelial human cancers.

**Tumor type**	**Site**	**Prognosis**	**Stage**	**Subcellular localization**	**Observations**	**References**
NSCLC	Serum	–	Advanced	n/a	Serum HMGB-1 negatively correlated with response to chemotherapy and survival (145)	(145, 146)
NSCLC	Tissue	–	Advanced	n/a (147); n/s (148)	Significant association observed between the gene expression levels of HMGB1 and MMP-9 (147); high expression of HMGB1 was closely related to the poor prognosis of patients with lung cancer (148)	(147, 148)
NSCLC	Tissue	+	Advanced (149); I-IV (150)	Mainly cytoplasm (149);	Low HMGB1 associated with poor immune activation	(149, 150)
BREAST	Tissue	+ (151)	I-IV (151); I-III (152) (153) (133); early (154)	Nucleus (155) (133); mainly cytoplasm (151); both (153)	Less differentiated carcinoma presented more diffused localization of HMGB1 in the nucleus (155); Cytoplasmic HMGB1 associated with small tumor size and early stages (151); no prognostic significance (152); cytoplasmic HMGB1 associated with TIL, but no prognostic significance (153) (156); post-chemotherapy increase in circulating HMGB1 correlated with better survival (154)	(133, 151–156)
BREAST	Serum	–	Advanced	n/a	High pre-chemotherapy HMGB1 levels predicted a later therapy response	(157)
CRC	Tissue	–	Advanced	Nucleus (155, 158)	Less differentiated carcinoma presented more diffused localization of HMGB1 in the nucleus (155); HMGB1 in the cytoplasm reduces infiltration of CD45^+^ cells (158); High HMGB1 in tumor tissue correlated with metastasis and lower DCs infiltration (159)	(155, 158–165)
CRC	Serum	–	Advanced and Early (166)	n/a	No correlation between serum HMGB1 and patient survival (166)	(166–168)
ESOPHAGEAL	Serum	–	I-IV	n/a	HMGB1 increment after neoadjuvant chemotherapy; Preoperative serum HMGB-1 may be associated to response to preoperative treatment	(169)
ESOPHAGEAL	Serum	+	I-IV	n/a	HMGB1 linked to immunogenic cell death	(170)
ESOPHAGEAL	Tissue	–	I-III	Both	HMGB1 expression positively correlated with expression of VEGF-C, lymph node metastasis, MLD and stage	(171)
PANCREATIC	Tissue	–	Advanced and Early	Mainly nucleus	The combination of HMGB1^+^/HMGB2^−^ expression linked to poor prognosis	(172)
PANCREATIC	Tissue	+	Advanced and Early	Mainly nucleus	Diminished nuclear and total cellular expression of HMGB1 in PDAC correlates with poor overall survival	(173)
PANCREATIC	Serum	–	Advanced and Early	n/a	HMGB1 as a potential diagnostic biomarker for PDAC (174); Circulating nucleosomes and HMGB1 as prognostic factors (175)	(174, 175)

*NSCLC, non-small cell lung carcinoma; n/a, not applicable; n/s, not specified; TIL, tumor infiltrating lymphocytes; DCs, dendritic cells; MMP-9, matrix metalloproteinase; VEGF-C, vascular endothelial growth factor C; MLD, micro-lymphatic vessel density*.

Although information available in the current literature can vary regarding the role, localization and clinical relevance of HMGB1, it is of extreme interest to explore its prognostic and biomarker potential, since it appears to be altered in so many human cancers (142). Also, some evidence present HMGB1 as a candidate predictive of therapy responsiveness in a plethora of treatment settings. However, it is yet to be determined where best to look–within the circulation or in tumor tissues–and if subcellular localization can offer any clinically relevant information.

### HMGB1 and miRNAs–an Unusual Liaison

One of the most extensively studied features of HMBG1 involves its ability to induce autophagy in situations of stress, including therapy, leading to drug resistance. Interestingly, miRNAs, short sequences that act as non-coding, post-translational regulators of multiple target genes, also seem to be regulators of this biologic phenomenon through direct modulation of HMGB1 expression. The role of individual miRNAs in controlling autophagy mediated by HMGB1 and their predicted targets in humans, according to the TargetScan or miRDB databases, are shown in Table 2.

**Table 2:** HMGB1-mediated autophagy is controlled by microRNAs.

**miR**	**Predicted targets (top 5)**	**Disease association**	**Observations**	**References**
miR-410-3p (http://www.targetscan.org/cgi-bin/targetscan/vert_71/targetscan.cgi?mirg=hsa-miR-410-3p)	NPPC DCTN6 CBFB TRAPPC3 ARFIP1	PDAC	Chemosensitization via inhibition of HMGB1-mediated autophagy	(180)
miR-34-a (http://www.targetscan.org/cgi-bin/targetscan/vert_71/targetscan.cgi?mirg=hsa-miR-34a-5p)	MDM4 HCN3 FAM76A SCN2B SYT1	AML, retinoblastoma	Chemosensitization via inhibition of HMGB1-mediated autophagy	(181, 182)
miR-142-3p (http://www.targetscan.org/cgi-bin/targetscan/vert_71/targetscan.cgi?mirg=hsa-miR-142-3p.1)	BOD1 WASL RHOBTB3 FAM114A1 MANBAL	AML	Chemosensitization via inhibition of HMGB1-mediated autophagy	(183)
miR-142-3p (http://www.targetscan.org/cgi-bin/targetscan/vert_71/targetscan.cgi?mirg=hsa-miR-142-3p.1)	BOD1 WASL RHOBTB3 FAM114A1 MANBAL	NSCLC	Chemosensitization via inhibition of HMGB1-mediated autophagy	(184)
miR-129-5p (http://www.targetscan.org/cgi-bin/targetscan/vert_71/targetscan.cgi?mirg=hsa-miR-129-5p)	CACNG2 IGIP CAMK2N1 YIPF5 LRRC4C	Breast	Chemosensitization via inhibition of HMGB1-mediated autophagy	(185)
miR-218 (http://www.targetscan.org/cgi-bin/targetscan/vert_71/targetscan.cgi?mirg=hsa-miR-218-5p)	TUB VOPP1 SGCZ TPD52 C3orf70	Endometrial	Chemosensitization via inhibition of HMGB1-mediated autophagy	(186)
miR-22 (http://www.targetscan.org/cgi-bin/targetscan/vert_71/targetscan.cgi?mirg=hsa-miR-22-5p)	PCDH15 DST ATP6V1G3 MINPP1 MAS1	Osteosarcoma	Chemosensitization via inhibition of HMGB1-mediated autophagy; Inhibition of proliferation, migration and invasion of tumor cells (187)	(187, 188)
miR-200c (http://www.targetscan.org/cgi-bin/targetscan/vert_71/targetscan.cgi?mirg=hsa-miR-200c-5p)	GNG13 SYT4 PGPEP1L LSMEM1 CPXCR1	NSCLC	Chemosensitization via inhibition of HMGB1-mediated autophagy	(189)
miR-129-2 (http://mirdb.org/cgi-bin/search.cgi?searchType=miRNA&full=mirbase&searchBox=MIMAT0004605)	BDKRB2 TMEM136 CCP110 SCN3B SEC14L1	Glioma	Chemosensitization via inhibition of HMGB1-mediated autophagy	(190)
miR-505 (http://www.targetscan.org/cgi-bin/targetscan/vert_71/targetscan.cgi?mirg=hsa-miR-505-5p)	CLEC2A SMIM6 PITPNM3 CFC1B AKT1S1	HCC	Inhibition of HMGB1-mediated DNA repair and inactivation of Akt pathway	(191)
miR-141 (http://www.targetscan.org/cgi-bin/targetscan/vert_71/targetscan.cgi?mirg=hsa-miR-141-5p)	DST CDR1 C1orf54 HMGB1 RSPH4A	Acute pancreatitits	Inhibition of HMGB1-mediated autophagy may decrease tissue injury *in vivo*	(192)

*PDAC, pancreatic ductal adenocarcinoma; AML, acute myeloid leukemia; NSCLC, non-small cell lung carcinoma; HCC, hepatocellular carcinoma; Human predicted targets retrieved from the TargetScan and miRDB databases*.

In PDAC pre-clinical models, expression of HMGB1 and miR-410-3p inversely correlate with responsiveness to gemcitabine and in patients, expression of this miRNA is associated with good prognosis. From an array of 30 genes, HMGB1 was the only gene under the control of miR-410-3P which was overexpressed in gemcitabine-resistant human PDAC cells. Importantly, it was demonstrated that overexpression of miR-410-3P inhibits formation of HMGB1-mediated LC3 puncta in gemcitabine-treated PDAC cells (180). Similar results were found in doxorubicin and cisplatin-treated osteosarcoma cells *in vitro*, where miR-22 regulates HMGB1-induced post-chemotherapy autophagy (188) and in paclitaxel-treated endometrial cancer cells where miR-218 plays this role (186). miR-34a has previously been identified as a tumor suppressor, downregulated in various cancers (193–195). In acute myeloid leukemia cells, miR-34a overexpression stimulates apoptosis through regulation of Bax and Bcl-2, in addition to inhibiting autophagy following treatment with all-trans retinoic acid (181). This autophagy limiting, apoptosis inducing property of miR-34a has also been demonstrated in retinoblastoma, where transfection with a miR-34a mimic enhanced *in vitro* sensitivity to vincristine, etoposide and carboplatin, in addition to increasing markers of DNA damage, ROS production and loss of mitochondrial membrane potential (182). In breast cancer, miR-129-5p direct regulation of HMGB1 and consequently of autophagy contributes to ameliorate radiosensitivity *in vitro* (185). Additionally, even though the effects on chemosensitivity were not evaluated, miR107 was found to be downregulated in human breast tumors and cell lines (196). This miR targets HMGB1 directly and regulates its autophagy inducing properties *in vitro*, while overexpression reduces tumorigenesis *in vivo*. miR-142-3p can both down-regulate expression of HMGB1 and increase signaling through the PI3K/Akt/mTOR pathway, decreasing post-chemotherapy autophagy in lung tumor cells *in vitro* (184). Additionally, miR-142-3p-overexpressing lung tumors have lower expression of HMGB1 and increased sensitivity to doxorubicin and cisplatin *in vivo*. In AML, miR-142-3p is also implicated in the direct regulation of HMGB1, inhibiting autophagy and enhancing drug sensitivity (183). Lower expression of miR-142-3p and higher expression of HMGB1 in PBMCs from pediatric AML patients is found.

In hepatocellular carcinoma (HCC) cell lines, miR-505 negatively regulates HMGB1, increasing doxorubicin cytotoxicity *in vitro* via enhanced caspase 3 activity, induction of DNA damage and decreased phosphorylation of Akt, a pathway known to be closely involved in drug resistance (191).

Some chemotherapeutic agents such as doxorubicin, mitoxantrone, oxaliplatin and bortezomib can elicit so-called “immunogenic cell death” (ICD). Markers of ICD include calreticulin CRT exposure on the cell membrane, secretion of ATP and HMGB1, which are often accompanied by increases in autophagic flux. In the context of chemoresistance, HMGB1 is detrimental because it prevents cells from dying (inhibiting apoptosis and promoting autophagy) whereas in ICD, HMGB1-induced autophagy is considered an immunogenic signal that will ultimately lead to tumor elimination. In this context, miR-27a controls CRT translocation and secretion of ATP and HMGB1 after treatment of colorectal cancer cells with ICD inducers (197). miR-27a low-expressing tumor cells released more HMGB1 into the extracellular space, displaying a more autophagic phenotype able to induce DCs phenotypical and functional maturation *in vitro*.

## A Teaser: HGF and HMGB1 as Reciprocally Regulated Hormonal Mediators in Cell Death

Although we usually consider apoptotic cell death as a “quiet death,” reparative proliferation of epithelia requires some form of communication to persisting cells that replication is in order. Interestingly, hepatocyte growth factor is released from apoptotic cells (198–203) and HMGB1, as detailed above, from necrotic and necroptotic cells (204). HGF release is associated with signaling through the Met receptor to upregulate CXCR4 expression (205–207). Interestingly, CXCR4 is an important receptor, as noted above, for SDF1/CXCL12/HMGB1 heterodimers that promote recruitment of inflammatory cells (67, 208–213). HMGB1 is released following tissue injury, forming a heteroduplex with CXCL12. Signaling through CXCR4 promotes response to injury, tissue regeneration and increase in cell cycling by promoting quiescent stem cell transition from G0 to GAlert. Most of the “reparative” program of tissues involved with wound healing and normal epithelial barrier function involves this pathway. In the setting of cancer, neoplastic epithelia exhibit an exaggerated program of tissue repair associated with premature and unscheduled cell death leading to a folie a' deux of DAMP release, reparative proliferation, and inhibition of immunity, something we refer to as the DAMP hypothesis to explain cancer and perhaps other chronic inflammatory states.

## Conclusions

Even though HMGB1 was first described as a nuclear protein, today we know that it is also one of the major cytokine-like mediators of inflammation and its pathological discontents, such as autoimmunity and cancer. HMGB1 is a highly context-dependent molecule, exerting various biologic effects depending on its partnering molecule, redox state, and subcellular location. Furthermore, HMGB1-mediated autophagy participates in important cellular processes such as cell fate decisions, chronicity of inflammatory responses and chemosensitivity to cancer-ablative drugs. While HMGB1, as a DAMP, can attract immune cells to the tumor site and engage receptors such as TLRs, culminating in immune activation, its presence in human tumor tissues and circulation is frequently associated with disease severity and progression. In autoimmunity, patient's HMGB1 levels increase in the active phase of disease, worsening inflammation and symptoms. Thus, it is of extreme importance to further elucidate underlying mechanisms involving HMGB1 signaling in pathology, in order to possibly one day use it as a therapeutic target, prognostic or even diagnostic biomarker in patients suffering from autoimmune disorders and cancer.

## Author Contributions

CG, GR, RB, and ML contributed to the elaboration of the manuscript.

### Conflict of Interest Statement

The authors declare that the research was conducted in the absence of any commercial or financial relationships that could be construed as a potential conflict of interest.
